# TIM3^+^CD8^+^ T细胞在非小细胞肺癌中心区和非中心区微环境中的表达及其临床意义

**DOI:** 10.3779/j.issn.1009-3419.2024.102.46

**Published:** 2024-12-20

**Authors:** Jiajuan WU, Shiying GUO, Leilei LV, Jiawei ZHAI, Yu SHEN, Cheng CHEN, Qiuxia QU

**Affiliations:** ^1^215006 苏州，苏州大学附属第一医院江苏省临床免疫研究所; ^1^Jiangsu Institute of Clinical Immunology; ^2^呼吸与危重症医学科; ^2^Department of Respiratory and Critical Medicine,the First Affiliated Hospital of Soochow University, Suzhou 215006, China

**Keywords:** 肺肿瘤, TIM3, PD-1, CD8^+^ T细胞, 肿瘤微环境, 免疫治疗, Lung neoplasms, TIM3, PD-1, CD8^+^ T cells, Tumor microenvironment, Immunotherapy

## Abstract

**背景与目的:**

免疫检查点抑制剂已成为非小细胞肺癌（non-small cell lung cancer, NSCLC）治疗的重要手段，但仍有部分患者未能从中获益，揭示参与免疫治疗的关键CD8^+^ T细胞亚群，并建立可靠的生物标志物至关重要。本研究旨在分析TIM3^+^CD8^+^ T细胞在NSCLC组织微环境中不同区域的表达，并探讨其作为新的生物标志物的价值。

**方法:**

收集NSCLC患者肿瘤组织样本，根据活检方式分为肿瘤中心和肿瘤非中心区域，利用流式细胞术分析TIM3^+^CD8^+^ T细胞的浸润及其表面程序性细胞死亡受体1（programmed cell death 1, PD-1）的表达，并和免疫治疗疗效作关联分析。

**结果:**

肿瘤组织非中心区域TIM3^+^CD^+^ T细胞的浸润水平显著高于肿瘤中心区域（*P*<0.0001），肿瘤直径≥3 cm或<3 cm亚组均呈现这种特征（*P*<0.01）；与TIM3^-^CD8^+^ T细胞相比，TIM3^+^CD8^+^ T细胞表达更高水平的PD-1（*P*<0.001），PD-1^+^TIM3^+^CD8^+^ T细胞更多浸润于肿瘤直径≥3 cm的肿瘤非中心区域（*P*<0.01）；免疫治疗临床应答者肿瘤非中心区域TIM3^+^CD8^+^ T细胞浸润水平显著低于无应答者，浸润程度越低，临床缓解程度越显著（*P*<0.01），而肿瘤中心区域TIM3^+^CD8^+^ T细胞与此无显著关联（*P*>0.05）；受试者工作特征（reciever operating characteristic, ROC）曲线显示肿瘤非中心区域TIM3^+^CD8^+ ^T细胞预测免疫治疗疗效的曲线下面积（area under the curve, AUC）达0.9375，显著高于肿瘤中心区域TIM3^+^CD8^+^ T细胞，也优于程序性细胞死亡配体1（programmed cell death ligand 1, PD-L1）[肿瘤阳性细胞比例分数（tumor proportion score, TPS）]预测能力。

**结论:**

TIM3^+^CD8^+^ T细胞在NSCLC组织微环境中的中心区和非中心区呈现区域性分布特征，位于肿瘤非中心区域微环境中该细胞群体的表达可作为预测免疫治疗疗效的潜在生物标志物。

## Abstract (en)

**Background and objective:**

One of the most important treatment modalities for non-small cell lung cancer (NSCLC) is immune checkpoint inhibitor. Nevertheless, a small percentage of patients do not respond well to these therapies, highlighting the significance of identifying important CD8^+^ T cell subsets for immunotherapy and creating trustworthy biomarkers. The purpose of this study is to assess the potential utility of TIM3^+^CD8^+^ T cells as new biomarkers by examining their expressions in various areas of the NSCLC tumor microenvironment.

**Methods:**

Based on biopsy techniques, tumor tissue samples were obtained from patients with NSCLC and categorized into tumor central and non-central regions. Using flow cytometry, the infiltration of TIM3^+^CD8^+^ T cells and the surface expression of programmed cell death 1 (PD-1) on these cells were examined, and their correlations with the effectiveness of immunotherapy were assessed.

**Results:**

The non-central region of tumor tissues had considerably larger infiltration of TIM3^+^CD8^+^ T lymphocytes compared to the non-central region (*P*<0.0001). This pattern was found in both subgroups with tumor diameters ≥3 cm or <3 cm (*P*<0.01). In comparison to TIM3^-^CD8^+^ T cells, TIM3^+^CD8^+^ T cells showed higher levels of PD-1 (*P*<0.001), with more PD-1^+^TIM3^+^CD8^+^ T cells invading the non-central region (*P*<0.01). Clinical responders to immunotherapy had considerably lower infiltration levels of TIM3^+^CD8^+^ T cells in the tumor non-central region compared to non-responders, with lower levels correlated with better clinical outcomes (*P*<0.01), while no correlation was identified in the tumor central region (*P*>0.05). According to reciever operating characteristic (ROC) curve analysis, TIM3^+^CD8^+ ^T cells in the tumor non-central region had an area under the curve (AUC) of 0.9375 for predicting the effectiveness of immunotherapy, which was considerably higher than that of TIM3^+^CD8^+^ T cells in the tumor central region and programmed cell death ligand 1 (PD-L1) [tumor proportion score (TPS)].

**Conclusion:**

In the tumor microenvironment of NSCLC, TIM3^+^CD8^+^ T cells show regional distribution patterns. The expression of this cell population in the non-central region of the tumor microenvironment may be a biomarker for predicting the effectiveness of immunotherapy.

肺癌是全球死亡率最高的恶性肿瘤，其中，非小细胞肺癌（non-small cell lung cancer, NSCLC）是主要的病理类型^[1]^。针对程序性细胞死亡受体1（programmed cell death 1, PD-1）及程序性细胞死亡配体1（programmed cell death ligand 1, PD-L1）的免疫检查点抑制剂的应用显著改变了NSCLC的临床实践^[2,3]^，但提升临床响应和构建稳健的生物标志物有待进一步深入研究。

肿瘤微环境（tumor microenvironment, TME）是一个复杂的生态系统^[4]^。研究^[5,6]^表明，异质性、区域性和时空性是TME的重要特征，但因受限于符合伦理的人体肿瘤区域性生物样本的获取制约了该领域的发展。T细胞免疫球蛋白和黏蛋白结构域3（T cell immunoglobulin and mucin-domain containing-3, TIM3）是一种I型跨膜糖蛋白，属于磷脂酰丝氨酸（phosphatidylserine, PS）受体家族^[7]^。TIM3最初在CD4^+^和CD8^+ ^T细胞中被发现，作为一种负性免疫检查点，其在多种免疫细胞上均有表达，包括1型辅助性T细胞（Th1）细胞、Th17细胞、调节性T（Treg）细胞和先天免疫细胞^[8]^，其结合其配体、半乳糖凝集素-9（galectin 9, Gal-9）、癌胚抗原相关细胞黏附分子1（carcinoembryonic antigen-related cell adhesion molecule 1, CEACAM-1）和高迁移率族蛋白B1（high mobility group box 1, FMGB1）介导T细胞耗竭，参与慢性病毒感染和肿瘤进展^[9,10]^。研究^[11]^发现，TIM3与PD-1在肿瘤浸润的CD8^+ ^T细胞中的共表达与肾细胞癌的不良预后相关。此外，在NSCLC及消化系统肿瘤中还发现血清中存在可溶性TIM3（soluble TIM3, sTIM3）形式^[12,13]^。

鉴于此，本研究旨在利用不同支气管镜活检技术进一步评估NSCLC组织TME中不同区域TIM3^+^CD8^+ ^T细胞的浸润水平，并评估其作为免疫治疗疗效生物标志物的价值，为优化肺癌免疫治疗提供新的数据。

## 1 资料与方法

### 1.1 研究对象

纳入苏州大学附属第一医院呼吸与危重症医学科于2020年7月至2023年12月期间收治的肺癌患者共61例，均经病理检查确诊，同时收集患者临床信息，包括年龄、性别、组织学亚型、肿瘤大小、临床治疗和转归。研究经苏州大学附属第一医院伦理委员会批准（No.2019-070、No.2024-465）。

### 1.2 组织活检

利用支气管镜针对肿瘤新生物表面进行取材（肿瘤非中心部位），利用经气管超声内镜引导针吸活检术（endobronchial ultrasound-guided transbronchial needle aspiration, EBUS-TBNA）取材（肿瘤中心部位）。所得标本4 °C保存，当天完成检测。

### 1.3 肺癌组织中TIM3^+^CD8^+ ^T细胞的检测

#### 1.3.1 肺癌组织单个核细胞的获取

将新鲜肿瘤组织置于1.5 mL不含血清的RPMI-1640培养基中，使用无菌剪刀、镊子将组织剪碎，随后加入2 µL的DNaseI酶（0.3 mg/mL）和500 µL的Liberase™ TL酶（0.2 mg/mL），使用移液枪吹打使其混匀后放入37 °C的培养箱中消化。30 min后，加入2 mL FACS（含1% FBS的PBS）终止消化，并通过70 µm细胞筛过滤，随后将其转移至15 mL离心管中，1800 rpm离心5 min，弃上清。加入适量的培养基将细胞重悬，稀释、计数，将单个核细胞密度调整至1×10^6^/mL。

#### 1.3.2 肿瘤组织中TIM3^+^CD8^+ ^T细胞的流式细胞术分析

在获得的单个核细胞悬液中加入相应抗体染色，4 °C避光染色25 min，染色方案：APC-Cyanine7-labeled anti-CD45 mAb（HI30）、FITC-labeled anti-CD8α mAb（RPA-T8）、PE-labeled anti-TIM3 mAb（F38-2E2），抗体均购自Biolegend公司（美国）。使用Cytoflex（Beckman）流式细胞仪进行多色流式检测，数据使用FlowJo软件（Tree Star）进行分析。采用FlowJo V10.8软件对所得流式数据进行分析并绘制流式代表图。

#### 1.3.3 肿瘤组织中TIM3^+^CD8^+ ^T细胞上PD-1分子的分析

APC-labeled anti-PD-1 mAb（EH12.2H7）购自Biolegend公司（美国），使用Cytoflex（Beckman）流式细胞仪分析TIM3^+^CD8^+ ^T细胞和TIM3^-^CD8^+ ^T细胞上PD-1分子的表达。

### 1.4 临床免疫治疗疗效评估

选取可测量的靶病灶，按照实体瘤疗效评价标准1.1版（Response Evaluation Criteria in Solid Tumors 1.1, RECIST1.1）来进行免疫治疗疗效评估，分为完全缓解（complete response, CR）、部分缓解（partial response, PR）、病情稳定（stable disease, SD）、疾病进展（progressive disease, PD）。客观缓解率（objective response rate, ORR）定义为CR和PR比例之和。免疫治疗缓解深度定义为和基线相比，靶病灶的最大直径缩小比例。

### 1.5 统计学分析

采用GraphPad Prism 8.0软件对数据进行统计学分析并绘制统计图。文中符合正态分布的计量资料使用Mean±SD表示，若数据不符合正态分布则以中位数（P25, P75）表示。非配对样本均值比较使用Student t检验，数据不服从正态分布的组间比较则进行Mann-Whitney U秩和检验。配对样本的非正态分布数据，采用Wilcoxon符号秩检验。利用Spearman相关进行相关性分析。对免疫治疗疗效有预测意义的因素绘制受试者工作特征（reciever operating characterisric, ROC）曲线评估其预测效能。在所有假设检验中，*P*<0.05时为差异具有统计学意义。

## 2 结果

### 2.1 患者特征

本研究共纳入肺癌患者61例，男性48例，女性13例，年龄≥65岁者36例，<65岁者25例；按组织学类型，鳞癌43例，腺癌18例；按原发肿瘤最大直径，≥3 cm者39例，<3 cm者22例；按肿瘤原发灶-淋巴结-转移（tumor-node-metastasis, TNM）分期，IIIA期以下者28例，IIIA期及以上者33例。

### 2.2 NSCLC组织TME中TIM3^+^CD8^+ ^T细胞浸润水平的区域特征

首先，研究建立了NSCLC组织中TIM3^+^CD8^+ ^T细胞的流式细胞术分析方法（图1A），并建立了针对NSCLC组织的不同区域部位的取材技术（图1B）。结果显示，肿瘤组织非中心区域TIM3^+^CD8^+ ^T细胞的浸润水平显著高于肿瘤中心区域[35.65%（16.43%, 53.58%）vs 4.26%（1.17%, 25.00%），*P*<0.0001，图1C]；进一步分析发现，无论是肿瘤直径≥3 cm[32.85%（10.50%, 51.13%）vs 1.82%（1.05%，26.20%），*P*<0.01，图1C]或<3 cm[46.2%（23.38%, 64.35%）vs 9.63%（2.13%, 23.08%），*P*<0.01，图1C]，TIM3^+^CD8^+ ^T细胞的浸润均表现出上述区域性差异。以上结果表明，NSCLC组织TME中TIM3^+^CD8^+ ^T细胞在不同区域（中心区和非中心区）呈现区域性分布特征，这在一定程度上反映了TME的免疫特性。

**图 1:**
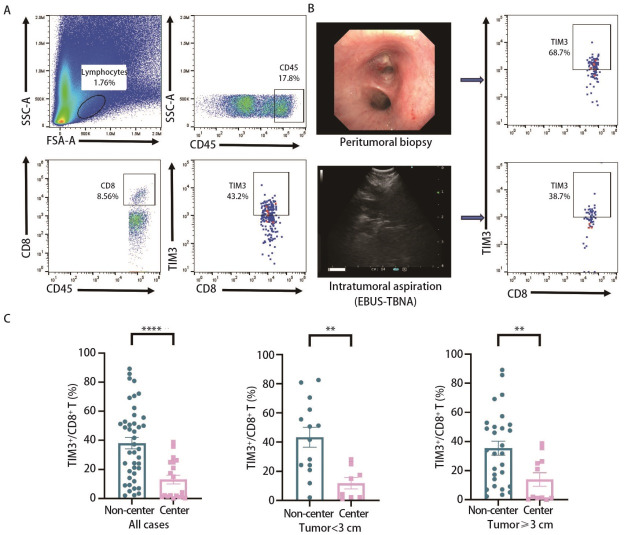
NSCLC肿瘤组织TIM3^+^CD8^+ ^T细胞的分析。A：NSCLC组织中TIM3^+^CD8^+ ^T细胞亚群的流式细胞术圈门策略；B：通过肿瘤表面活检和EBUS-TBNA获取分别代表非中心区域和中心区域的肿瘤组织；C：肿瘤组织非中心区域TIM3^+^CD8^+ ^T细胞的浸润水平显著高于肿瘤中心区域，肿瘤直径≥3 cm和<3 cm亚组均呈现这种特征。数据使用中位数（P25, P75）表示，使用Mann Whitney U检验，**P<0.01，****P<0.0001。

### 2.3 PD-1主要表达于TIM3^+^CD8^+ ^T细胞

本研究进一步对肿瘤浸润性TIM3^+^CD8^+ ^T细胞上PD-1的表达差异进行分析，圈门策略见图2A。结果显示，与TIM3^-^CD8^+ ^T细胞相比，TIM3^+^CD8^+ ^T细胞表达更高水平的PD-1[33.30%（13.25%, 54.90%）vs 21.55%（5.95%, 40.25%），*P*<0.001，图2B]；进一步分析发现，仅在肿瘤直径≥3 cm的亚组中观察到了PD-1^+^TIM3^+^CD8^+ ^T细胞更多浸润于肿瘤非中心区域[33.30%（10.00%, 55.40%）vs 25.00%（9.11%, 49.20%），*P*<0.01，图2C]，表明PD-1^+^TIM3^+^CD8^+ ^T细胞亚群可能在肿瘤侵袭生长过程中发挥更多的作用。

**图 2:**
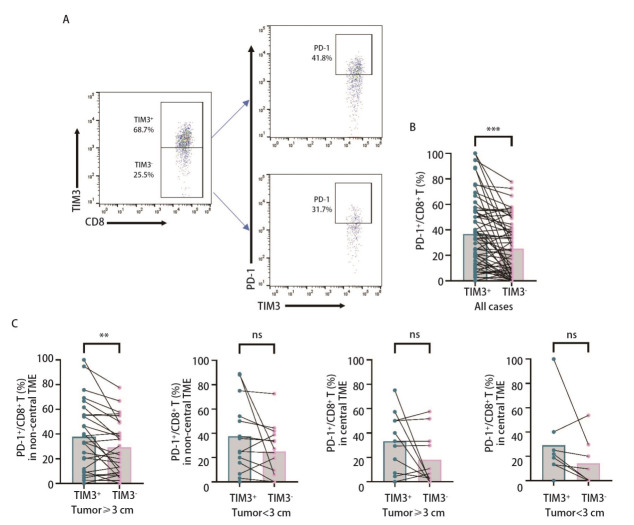
PD-1在TIM3^+^CD8^+ ^T细胞中的表达。A：PD-1在TIM3^+^CD8^+ ^T细胞和TIM3^-^CD8^+ ^T细胞亚群中的分析策略；B：与TIM3^-^CD8^+ ^T细胞相比，TIM3^+^CD8^+ ^T细胞中PD-1的表达更高；C：PD-1^+^TIM3^+^CD8^+^ T细胞仅在肿瘤直径≥3 cm的非中心区域表达更高。数据使用中位数（P25, P75）表示，Wilcoxon符号秩检验， **P<0.01，***P<0.001。

### 2.4 TIM3^+^CD8^+ ^T细胞和抗PD-1治疗缓解深度的关联

15例初治鳞癌患者和7例初治腺癌患者（经二代测序后未发现敏感性驱动基因改变）接受了含抗PD-1抗体的治疗（免疫单药8例，免疫联合化疗14例）。完成2个治疗周期后疗效评估显示，ORR为54.5%，PR 12例。分析显示，在22例患者中，免疫治疗ORR者TIM3^+^CD8^+ ^T细胞浸润水平低于无应答者[22.70%（15.55%, 33.40%）vs 42.80%（24.35%, 59.75%），*P*<0.05，图3A]。进一步分析显示，免疫治疗ORR者肿瘤非中心区域TIM3^+^CD8^+ ^T细胞浸润水平显著低于无应答者[27.90%（18.20%, 36.48%）vs 54.05%（45.43%, 72.53%），*P*<0.01，图3B]，而肿瘤中心区域的TIM3^+^CD8^+ ^T细胞浸润水平与此无显著关联[17.50%（5.40%, 23.75%）vs 22.50%（13.63%, 33.25%），*P*>0.05，图3C]。

**图 3:**
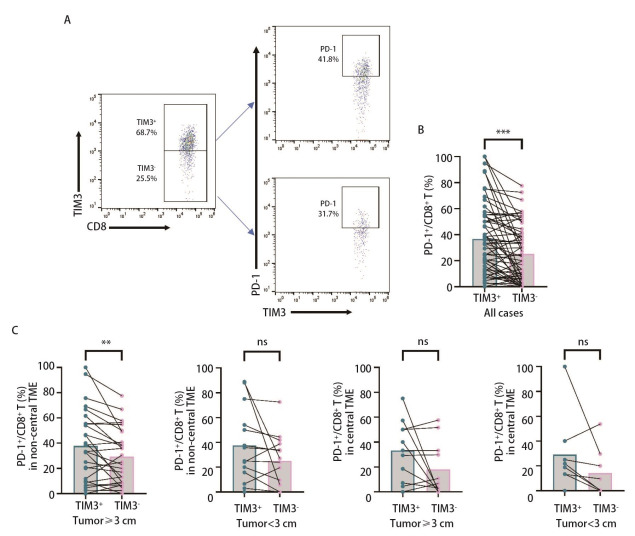
基于靶病灶最大直径变化比例建立瀑布图，采用Spearman相关分析评估TIM3^+^CD8^+ ^T细胞浸润水平与抗PD-1治疗后肿瘤缓解深度之间的关系。A：不加区分肿瘤中心和非中心区域时，TIM3^+^CD8^+ ^T细胞和抗PD-1免疫治疗疗效的关联分析；B：肿瘤非中心区域TIM3^+^CD8^+ ^T细胞浸润水平和抗PD-1免疫治疗疗效的关联分析；C：肿瘤中心区域TIM3^+^CD8^+ ^T细胞浸润和抗PD-1免疫治疗疗效的关联分析。*P<0.05，**P<0.01。

计算每例患者目标病灶直径相对于基线的百分比变化并生成瀑布图（图3）。在未加区分肿瘤中心和非中心区域时，TIM3^+^CD8^+ ^T细胞未表现出对免疫治疗的预测能力（r=0.2943，*P*>0.05，图3A）。亚组分析显示，在12例应答者中有9例患者的肿瘤非中心区域TIM3^+^CD8^+ ^T细胞比例低于cut-off值（*P*<0.01，图3B），且TIM3^+^CD8^+ ^T细胞浸润程度越低，抗PD-1治疗临床缓解程度越显著（r=0.5516，*P*<0.05，图3B）；而肿瘤中心区域的TIM3^+^CD8^+ ^T细胞未表现出类似的临床预测能力（r=0.3095，*P*>0.05，图3C）。上述结果提示，肿瘤非中心部位的TIM3^+^CD8^+ ^T细胞是免疫治疗响应的关键细胞群体，具有更高的潜力和临床应用价值的预测免疫治疗应答的生物标志物。

### 2.5 TIM3^+^CD8^+ ^T细胞作为免疫治疗生物标志物的分析

进一步构建了ROC曲线，如图4A和4B，当选择肿瘤非中心区域TIM3^+^CD8^+ ^T细胞占比48.75%作为cut-off值时，预测免疫治疗疗效的特异度为83.33%，敏感度为100.00%，曲线下面积（area under the curve, AUC）达0.9375，优于PD-L1[肿瘤阳性细胞比例分数（tumor proportion score, TPS）]预测能力。而当选择肿瘤中心区域TIM3^+^CD8^+ ^T细胞占比25.60%作为cut-off值时，预测疗效的特异度仅为50.00%，敏感度为100.00%，对应的AUC仅为0.6875。

**图 4:**
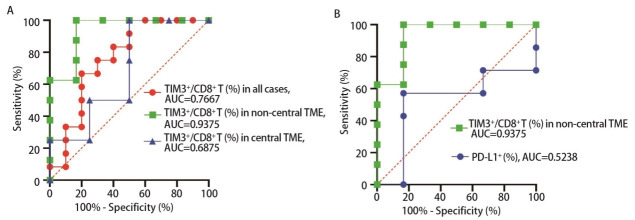
人肺癌组织中TIM3^+^CD8^+ ^T细胞对PD-1抗体疗效ROC曲线。A：肿瘤非中心区域（绿色）、中心区域（蓝色）、未区分肿瘤中心和非中心区域（红色）浸润TIM3^+^CD8^+ ^T细胞水平预测免疫治疗疗效的ROC曲线分析；B：肿瘤非中心区域（绿色）浸润TIM3^+^CD8^+ ^T细胞水平和PD-L1（TPS）（蓝色）预测免疫治疗疗效能力的比较。

## 3 讨论

目前，一些生物标志物如PD-L1表达和肿瘤突变负荷（tumor mutational burden, TMB）已被报道可用于预测免疫检查点抑制剂对NSCLC的疗效，但这些指标仍存在一定的局限性^[14,15]^。肿瘤浸润淋巴细胞（tumor infiltrating lymphocytes, TILs）的浸润水平及空间分布逐渐成为癌症免疫治疗领域的重要研究方向，并被认为是一种更有潜力的生物标志物^[16,17]^。

TME是癌症的一个重要组成部分，包括T细胞、B细胞和自然杀伤细胞等成分^[18]^。在肿瘤的发生、发展及抗肿瘤治疗过程中，TME的组成表现出显著的异质性，这种免疫异质性不仅存在于不同肿瘤类型之间，还体现在时间和空间的动态变化中^[19,20]^。鉴于此，本研究首先构建了基于支气管镜活检技术的NSCLC TME中心和非中心区域的取材方法。结果显示，无论肿瘤直径≥3 cm还是<3 cm，TIM3^+^CD8^+ ^T细胞亚群在肿瘤非中心区域的浸润水平均显著高于肿瘤中心区域。这一发现提示，TIM3^+^CD8^+ ^T细胞在NSCLC中不仅广泛分布，还呈现出显著的区域性特征，特别是其在肿瘤非中心区域的富集，这种区域性分布可能与TME中免疫抑制信号的局部调控密切相关。之前有研究^[21,22]^发现，在胃癌TME中，T细胞的耗竭显著削弱了其抗肿瘤作用，TME通过抑制免疫应答来阻碍抗肿瘤免疫反应，加速肿瘤的进展。此外，研究^[23]^还表明，在乳腺癌中，肿瘤中心区域的细胞通常表达与增殖较弱和静态表型相关的标志物，而肿瘤非中心区域的细胞则上调与侵袭和增殖相关的标志物，从而促进肿瘤向周边组织及血管的侵袭和转移。这些发现与本研究中观察到的TIM3^+^CD8^+ ^T细胞在肿瘤非中心区域显著富集的现象一致，进一步表明，肿瘤非中心区域的特异性微环境可能在调控免疫细胞的分布和功能中发挥了关键作用。

既往研究^[24]^表明，CD8^+ ^T细胞耗竭通常发生在TME中，表现为效应功能的进行性丧失和抑制性受体（如PD-1、TIM3等）的高表达。已有研究^[25]^证明PD-1和TIM3共表达增加与胆囊癌的不良预后和免疫微环境异质性密切相关。本研究结果显示，与TIM3^-^CD8^+ ^T细胞相比，TIM3^+^CD8^+ ^T细胞表达更高水平的PD-1，PD-1^+^TIM3^+^CD8^+ ^T细胞更多浸润于肿瘤非中心区域。这一现象在肿瘤直径≥3 cm的患者肿瘤非中心区域尤为显著，而在肿瘤直径<3 cm的患者中则未观察到类似趋势。这一结果提示，随着肿瘤体积的增大，免疫抑制信号可能逐步增强，尤其在肿瘤非中心区域的积累更加明显，这可能导致CD8^+ ^T细胞的功能进一步耗竭。

基于以上发现，本研究重点分析了NSCLC组织微环境中不同区域TIM3^+^CD8^+^ T细胞作为免疫治疗生物标志物的潜在价值。虽然研究已表明PD-L1表达、TMB可以作为NSCLC免疫治疗的预测性生物标志物^[26]^，然而，由于TME的高度异质性，这些标志物在预测免疫治疗效果方面仍存在一定局限性^[27]^。本研究结果显示，在肿瘤非中心区域，抗PD-1治疗临床应答者和与无应答者之间的TIM3^+^CD8^+ ^T细胞浸润水平存在差异，TIM3^+^CD8^+ ^T细胞浸润程度越低，抗PD-1治疗的临床缓解程度越显著，而肿瘤中心区域的TIM3^+^CD8^+ ^T细胞未表现出类似的临床预测能力。更为重要的是，肿瘤非中心区域TIM3^+^CD8^+ ^T细胞预测效能优于PD-L1（TPS）。值得注意的是，一项针对多种恶性肿瘤的meta分析^[28]^表明，TIM3的表达水平通常与较高的肿瘤分期和淋巴结转移的侵袭性密切相关。在结直肠癌患者的肿瘤组织中，TIM3表达水平显著增加，其上调与较差的患者预后紧密相关^[29]^。此外，在肝细胞癌中，TIM3^+^肿瘤浸润细胞的数量与患者生存率呈负相关^[30]^。在胶质母细胞瘤中，TIM3的高表达促进了TME中的免疫抑制，进一步加剧了患者的预后不良^[31]^。这些研究结果表明，TIM3的表达不仅与肿瘤的恶性程度相关，还可能成为预测患者预后和免疫治疗反应的重要生物标志物，进一步支持了TIM3^+^CD8^+ ^T细胞作为潜在临床预测标志物的价值。

然而，虽然位于NSCLC肿瘤非中心区域微环境中TIM3^+^CD8^+ ^T细胞群体的表达可作为预测抗PD-1治疗疗效的生物标志物，然而，其潜在的调控机制尚未完全清楚。例如，TIM3在CD8^+ ^T中的高表达如何影响抗PD-1治疗的效果，以及TIM3^+^CD8^+ ^T细胞作为生物标志物的具体作用机制仍需深入研究。

综上所述，本研究通过利用支气管镜活检技术评估NSCLC组织微环境中不同区域TIM3^+^CD8^+ ^T细胞的浸润水平及临床意义，确认了TIM3^+^CD8^+ ^T细胞在NSCLC TME中呈现区域性分布特征，位于肿瘤非中心区域的该细胞群体的表达可作为预测免疫治疗疗效的良好生物标志物，为进一步探索抗PD-1治疗耐药机制提供了新的研究思路。
